# Patterns of Natural and Human-Caused Mortality Factors of a Rare Forest Carnivore, the Fisher (*Pekania pennanti*) in California

**DOI:** 10.1371/journal.pone.0140640

**Published:** 2015-11-04

**Authors:** Mourad W. Gabriel, Leslie W. Woods, Greta M. Wengert, Nicole Stephenson, J. Mark Higley, Craig Thompson, Sean M. Matthews, Rick A. Sweitzer, Kathryn Purcell, Reginald H. Barrett, Stefan M. Keller, Patricia Gaffney, Megan Jones, Robert Poppenga, Janet E. Foley, Richard N. Brown, Deana L. Clifford, Benjamin N. Sacks

**Affiliations:** 1 Integral Ecology Research Center, Blue Lake, California, United States of America; 2 Mammalian Ecology and Conservation Unit, Veterinary Genetics Laboratory, University of California Davis, Davis, California, United States of America; 3 California Animal Health and Food Safety Laboratory System, University of California Davis, California, United States of America; 4 University of California Davis, School Veterinary Medicine, Davis, CA, United States of America; 5 Wildlife Department, Hoopa Tribal Forestry, Hoopa, California, United States of America; 6 Pacific Southwest Research Station-Sierra Nevada Research Center, United States Forest Service, Fresno, California, United States of America; 7 Wildlife Conservation Society, Hoopa, California, United States of America; 8 Sierra Nevada Adaptive Management Project, University of California, Berkeley, California, United States of America; 9 Department of Pathology, Microbiology and Immunology, University of California Davis, Davis, California, United States of America; 10 Department of Wildlife, Humboldt State University, Arcata, California, United States of America; 11 Wildlife Investigations Laboratory, California Department of Fish and Wildlife, Rancho Cordova, California, United States of America; 12 Department of Population Health and Reproduction, University of California Davis, Davis, California, United States of America; University of Florida, UNITED STATES

## Abstract

Wildlife populations of conservation concern are limited in distribution, population size and persistence by various factors, including mortality. The fisher (*Pekania pennanti*), a North American mid-sized carnivore whose range in the western Pacific United States has retracted considerably in the past century, was proposed for threatened status protection in late 2014 under the United States Endangered Species Act by the United States Fish and Wildlife Service in its West Coast Distinct Population Segment. We investigated mortality in 167 fishers from two genetically and geographically distinct sub-populations in California within this West Coast Distinct Population Segment using a combination of gross necropsy, histology, toxicology and molecular methods. Overall, predation (70%), natural disease (16%), toxicant poisoning (10%) and, less commonly, vehicular strike (2%) and other anthropogenic causes (2%) were causes of mortality observed. We documented both an increase in mortality to (57% increase) and exposure (6%) from pesticides in fishers in just the past three years, highlighting further that toxicants from marijuana cultivation still pose a threat. Additionally, exposure to multiple rodenticides significantly increased the likelihood of mortality from rodenticide poisoning. Poisoning was significantly more common in male than female fishers and was 7 times more likely than disease to kill males. Based on necropsy findings, suspected causes of mortality based on field evidence alone tended to underestimate the frequency of disease-related mortalities. This study is the first comprehensive investigation of mortality causes of fishers and provides essential information to assist in the conservation of this species.

## Introduction

Identifying the factors limiting imperiled wildlife populations requires an understanding of all influences affecting population growth and persistence. The geographic range of the fisher, (*Pekania pennanti*), a medium-sized mesocarnivore that inhabits northern North America, has contracted significantly over the past century [1, 2]. Several factors potentially explain this contraction, including trapping and habitat alteration associated with fire management and logging throughout the early 1900s [1–4].

Recent conservation efforts, such as reintroductions and forest restoration to improve habitat, have helped to increase the fisher’s range from a range-wide low of 43% back to 68% of its historical range [1]. However, recent expansions were concentrated primarily in the central and eastern portions of the fisher’s range. Fisher populations in the Pacific states (Washington, Oregon and California) currently occupy only 21% of their historic distribution in this region and have not expanded, even in some regions with ample available suitable habitat and limited forest fragmentation [1, 2]. Fishers were extirpated from the state of Washington and northern and central Oregon prior to reintroductions to these regions from northern and eastern populations [2–4]. Isolation and failure of population expansion in this portion of their range has prompted the United States Fish and Wildlife Service (USFWS) to deem these populations in these Pacific states a West Coast Distinct Population Segment (DPS) and propose them for listing under the US Endangered Species Act as a threatened species [4]. In 2015, the California Department of Fish and Wildlife listed the southern Sierra Nevada population of fishers, but not the northern California population, threatened under the California Endangered Species Act.

California contains two genetically and geographically distinct native populations of fishers within this DPS [2, 5–7]. The northern California population inhabits the coastal and southern Cascade mountain ranges and is the larger of two California populations. The southern Sierra Nevada population is considerably smaller, thought to contain approximately 300 individuals with fewer than 120 breeding females [2, 8].

The USFWS considers five potential limiting factors as merits for listing: 1) destruction or modification of the habitat or species’ range; 2) overutilization for commercial, recreational, scientific or educational purposes; 3) disease or predation; 4) the inadequacy of existing regulatory mechanisms; or 5) other natural or manmade factors affecting its continued existence. Investigation into the frequencies of different causes of mortality can lend information to several of these concerns, most specifically factors 2, 3, and 5. Though several studies on western fisher populations have included descriptions of isolated cases of mortality for fishers [9–11], a systematic, large-scale investigation into cause-specific mortality as determined through full necropsies has not been conducted, specifically within the West Coast DPS [2, 12, 13]. Since 2004, several long-term studies of the California fisher populations have been initiated investigating demographics, habitat utilization, and mortality, and we took the opportunity to investigate fisher mortality across projects for a more comprehensive examination throughout California.

The objectives of the present study were to document causes of mortality in two distinct populations of California fishers using necropsy, histology, toxicology and molecular methods and to investigate demographic, temporal, spatial, and health-related patterns of the specific causes of mortality.

## Methods

All procedures involving animals were approved by the University of California, Davis, Institutional Animal Care and Use Committee (Protocol No. 16551) and state scientific collecting and salvage permits issued by the California Department of Fish and Wildlife (#SC-7304). Permission to conduct research at Hoopa Tribal Lands was granted by the Hoopa Tribal Council and Chief Wildlife Forestry Branch manager. Permission to conduct this research on United States Forest Service lands was provided by the Pacific Southwest US Forest Service Research Station.

### California Fisher Project areas and sampling

Fishers were collected through three long-term projects in California (Gabriel et al. 2012b), including one on the northern California population (Hoopa Valley Reservation Fisher Project, HVRFP) and two in the southern Sierra Nevada: the Sierra Nevada Adaptive Management Project (SNAMP), and the USFS Kings River Fisher Project (KRFP). The HVRFP project area was located in northwestern California on the Hoopa Reservation and adjacent private lands and federal United States Forest Service (USFS) public lands. The HVRFP personnel monitored fishers from the ground using telemetry approximately 1–2 times per week (J. Mark Higley, Hoopa Tribal biologist, personal communication). Both southern Sierra Nevada fisher projects were conducted on the Sierra National Forest in the northern and central portions of this population’s range. Fishers from the SNAMP project were located 3–6 times per week via aerial telemetry (Rick Sweitzer, SNAMP biologist, personal communication), while the KRFP personnel located each fisher via ground telemetry 2–3 times per week (Craig Thompson, USFS biologist, personal communication).

In all projects, fishers were captured in box traps (model 207, Tomahawk Live Trap Company, Tomahawk, Wisconsin, USA) modified with plywood cubby boxes to minimize environmental stressors [14, 15]. Each fisher was fitted with a VHF radio-collar and monitored via radio-telemetry. Radio-collars were equipped with activity or mortality sensors [16]. Inactivity on two consecutive location attempts separated by more than 24 hours or a single mortality signal from telemetry collars prompted attempts to recover carcasses as soon as was practical. When a fisher carcass was recovered, project biologists identified and recorded a suspected cause of mortality. Field based mortality determinations were constructed from evidence found at the immediate mortality site (predator tracks, nearby roadway, etc.) and the condition of the carcass (puncture wounds, cached carcass, etc.). Recovered fisher carcasses were stored in a -20°C freezer until further analysis. Fishers were subject to a complete necropsy performed by a board-certified veterinary pathologist specializing in wildlife at the University of California, Davis (UCD) Veterinary Medical Teaching Hospital or the California Animal Health and Food Safety Laboratory System (CAHFS) on the UCD campus. Additionally, any uncollared fishers that were collected opportunistically from the field within or near project areas were necropsied.

For each fisher carcass, age was determined by pulp-cavity closure or enumeration of cementum annuli of an upper premolar [17]. Fishers were classified as kits if they were altricial and dependent on mothers-milk for nourishment (roughly ≤10 weeks), juveniles if weaned and <12 months of age, sub-adults when between 12–24 months of age, and adults when ≥24 months of age [17].

Ancillary diagnostic testing was performed based on gross and histologic findings and consisted of molecular diagnostic tests to confirm a viral etiology [18], toxicological screening of selected tissues [12], forensic genetic tests of swabbed ante-mortem bite wounds to identify species of predators [19], and serology to determine exposure to three carnivore pathogens: canine distemper virus (CDV), canine parvovirus-2 (CPV), and *Toxoplasma gondii* [17].

Serological assessment and titer cutoffs were performed via indirect fluorescent-antibody (IFA) assays on uncoagulated blood collected by sterile cardiac puncture [17, 20, 21]. For both CDV and *T*. *gondii*, detection of both antibody isotype IgG, which persists for extended periods, and the short-duration antibody isotype IgM was used, while only detection of isotype IgG was used for CPV [22]. Isotype IgM was utilized for selected pathogen assays since recent or acute infections from these pathogens may predispose individuals to certain causes of mortality [23] [24, 25].

Predation was considered the cause of mortality if ante-mortem hemorrhage was observed and associated with bite, claw or talon wounds [19]. In addition, we followed up visible field signs of predation for which ante-mortem hemorrhaging could not be determined (e.g., due to consumption by the predator) with forensic DNA testing of tissue around putative bite wounds or tooth marks [19]. Mortalities were classified as “natural disease” if they exhibited clinically significant infectious (bacterial, viral, parasites, etc.) or non-infectious (malignant neoplasia, nutritional deficiency, etc.) factors that were considered by the pathologist to represent the primary cause of death [26–28]. Mortalities were classified as “poisoning” if an individual had acute clinically significant signs of toxicosis associated with toxicant exposure (e.g. carbamate, anticoagulant rodenticide). Fishers that died directly from anthropogenic factors (e.g. anesthesia, entrapment in human-structures) were classified as “human-caused.” Vehicular strike was considered to be the cause of death when carcasses were recovered on or near roads combined with evidence of blunt trauma. If a fisher carcass had insufficient tissues for a necropsy, severe autolysis or a lack of forensic evidence, its cause of death was classified as unknown.

### Statistical analysis

Statistical analyses were performed using R studio version 0.98.507 and the “mlogit” package [29, 30, 31]. A kappa statistic for test agreement was calculated to assess the strength of test agreement between field biologist-suspected cause of death and necropsy-confirmed cause of death [32]. We used a multinomial logistic regression model to assess the effects of several variables on the relative frequencies of cause-specific mortality which consisted of “natural disease,” “poisoning,” “predation,” and “human-caused,” however, we excluded unknown causes. We pooled human-caused mortalities with those from vehicular strike as “vehicular/ human” for modeling due to small sample sizes and the common anthropogenic source of mortality. We used two different data sets for modeling. The first included 136 radio-collared and uncollared fisher mortalities documented between 2007 and 2014 for which cause of mortality was known. We built models using all possible combinations of 1–5 variables which included population, sex, age class, season and year. The second data set included 72 fisher mortalities pulled from the previous group for which exposure status for both anticoagulant rodenticides and the three pathogens was known; no demographic parameters were used for this model. Models were built using all possible combinations of 1–8 variables which included IgG and IgM titers to CDV and *T*. *gondii*, IgG titer to CPV, exposure to AR, and the total number of AR detected. The latter two variables were not used in the same model to avoid multicollinearity among variables.

We employed an information-theoretic approach to identify the most parsimonious models [33] relating demographic parameters and disease and toxicant exposure parameters to cause of mortality. We calculated the Akaike Information Criteria score corrected for small sample sizes (AICc;[33]) for each model and compared the scores among competing models. We considered as final models those with ∆AICc < 2. The model was built using the outcome categories “predation,” “poisoning” and “natural disease” as the reference groups in three separate analyses, resulting in odds ratios (OR) for “predation vs. disease,” “predation vs. poisoning,” “predation vs. human-caused,” “poisoning vs. disease,” “poisoning vs. human-caused” and “disease vs. human-caused.” Model coefficients were estimated using the maximum-likelihood method [34].

## Results

A total of 167 fishers was collected for necropsy and/ or forensic examination from both California populations during 2007–2014 of which there were 105 adults (63%), 32 sub-adults (19%), 26 juveniles (16%) and 4 kits (2%). Males composed 44% (n = 73) and females 56% (n = 94) of all fisher mortalities. The necropsied population included 123 fishers (73%) which had adequate preservation and sufficient tissues for necropsy submittal. Of the remaining 44 fishers, 34 had suspected predator-inflicted wounds with insufficient tissues for necropsy and were submitted solely for molecular forensic examinations, while the remaining ten fisher carcasses (seven southern Sierra Nevada and three northern California) were too autolyzed for any examination.

Fifty-two (31%) of the fishers were from the northern California population while 115 (69%) were from the southern Sierra Nevada population (Table 1). Of the 163 adult, subadult or juvenile fishers (the four kits were excluded), 156 were radio-collared and seven were collected opportunistically, including three from northern California and four from the Sierra Nevada populations. Numbers of carcasses available for analysis were similar across years providing an approximately balanced multiannual data set (Table 1).

**Table 1:** Comparison of sex, age class, year of death, season of death and necropsy-determined cause of mortality for 167 fishers (*Pekania pennanti*) from two isolated populations, southern Sierra Nevada (South Sierra) and northern California (North CA). These data include both collared and uncollared fishers of all age classes.

		South Sierra (N = 115)		North CA (N = 52)	
Characteristic		n	%	n	%
*Sex*					
	Male	52	45.2	21	40.4
	Female	63	54.8	31	59.6
*Age Class*					
	Kit	3	2.6	1	1.9
	Juvenile	25	21.7	1	1.9
	Sub-Adult	21	18.3	11	21.2
	Adult	66	57.4	39	75.0
*Year of Death*					
	2007	6	5.2	7	13.5
	2008	13	11.3	6	11.5
	2009	25	21.7	3	5.8
	2010	21	18.3	6	11.5
	2011	20	17.4	8	15.4
	2012	11	9.6	10	19.2
	2013	15	13.0	5	9.6
	2014	4	3.5	7	13.5
*Season of Death*					
	Spring	52	45.2	26	50.0
	Summer	26	22.6	9	17.3
	Fall	16	13.9	9	17.3
	Winter	21	18.3	8	15.4
*Cause of Death*					
	Predation	67	58.3	23	44.2
	Natural Disease	16	13.9	9	17.3
	Poisoning	6	5.2	7	13.5
	Vehicular Strike	7	6.1	3	5.8
	Human	1	0.9	1	1.9
	Undetermined	18	15.6	9	17.3

### Necropsy-determined causes of mortality

**Fig 1:**
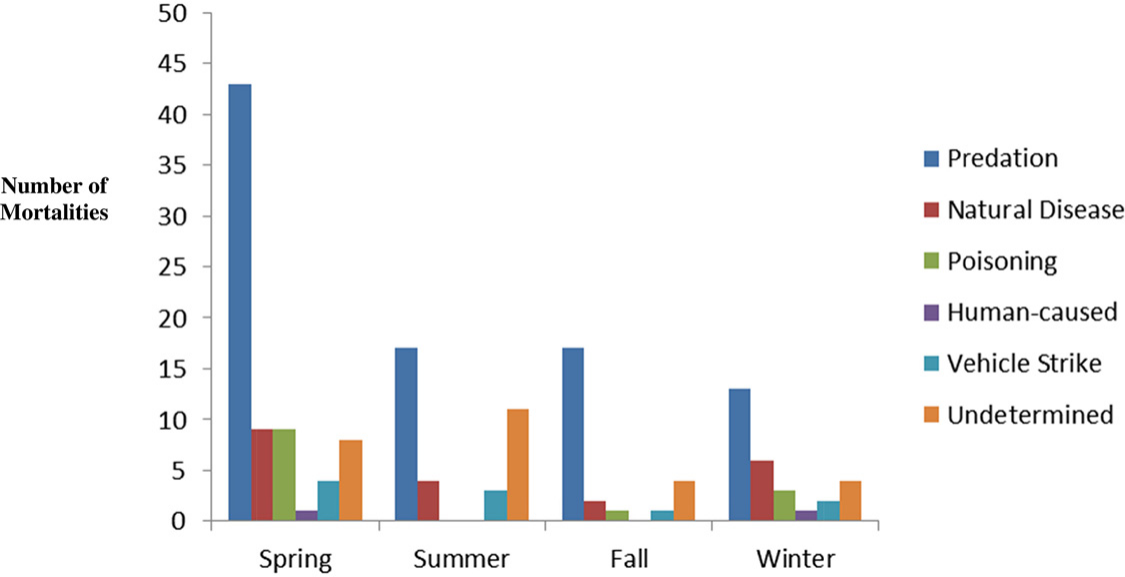
Contributions of each necropsy-determined cause of mortality confirmed by full necropsy and/ or forensic analysis over the seasons for California fisher (*Pekania pennanti*) populations in northern California and the southern Sierra Nevada. Data were combined from 2007 to 2014 (n = 167).

Confirmation of mortalities was based on necropsy and forensic examination and grouped into six categories based on our results: predation, natural disease, poisoning, vehicular strike, human-caused (other than vehicular strike) and unknown. We excluded fishers that were opportunistically collected due to vehicle strike (n = 7), kits recovered from dens (n = 4), and necropsied fishers whose cause of mortality was undetermined (n = 27) in order to more accurately represent the relative frequencies of different causes of mortality in the fisher populations. Of the 129 collared fishers for which cause of death was determined, predation was the highest contributing source of mortality (70%, n = 90), followed by natural disease (16%, n = 21), poisoning (10%, n = 13), vehicular strike (2%, n = 3) and human-caused (2%, n = 2) (Table 2, Fig 1).

**Table 2:** Necropsy-determined cause-specific mortality frequencies for fishers (*Pekania pennanti*) by sex, age, year and season from fisher populations in northern California and southern Sierra Nevada. Data were combined from 2007 to 2014 (n = 136). These data include 7 uncollared fishers discovered opportunistically dead due to vehicle strike so relative frequency of vehicle-related deaths may be overrepresented.

			Necropsy-determined Cause of Mortality							
		Total (n = 136)	Predation (n = 90)		Disease (n = 21)		Poisoning (n = 13)		Vehicular/Human (n = 12)	
Characteristic		n	n	%	n	%	n	%	n	%
*Population*										
	North Coast	42	23	54%	8	19%	7	17%	4	10%
	S. Sierra	94	67	71%	13	14%	6	6%	8	9%
*Sex*										
	Female	78	59	76%	11	14%	2	3%	6	8%
	Male	58	31	53%	10	17%	11	19%	6	10%
*Age*										
	Juvenile	22	17	77%	3	14%	1	5%	1	5%
	Sub-Adult	30	18	60%	7	23%	2	7%	3	10%
	Adult	84	55	65%	11	13%	10	12%	8	10%
*Year*										
	2007	12	8	67%	3	25%	0	0%	1	8%
	2008	17	9	53%	2	12%	1	6%	5	29%
	2009	25	12	48%	9	36%	1	4%	3	12%
	2010	19	15	79%	0	0%	3	16%	1	5%
	2011	22	19	86%	2	9%	0	0%	1	5%
	2012	17	10	59%	3	18%	3	18%	1	6%
	2013	17	12	70%	1	6%	4	24%	0	0%
	2014	7	5	71%	1	14%	1	14%	0	0%
*Season*										
	Spring	66	43	65%	9	14%	9	14%	5	8%
	Summer	24	17	71%	4	17%	0	0%	3	13%
	Fall	21	17	81%	2	10%	1	5%	1	5%
	Winter	25	13	52%	6	24%	3	12%	3	12%

### Predation

Of the 90 fishers that died from predation, necropsy examination confirmed 58 predation events. The remaining 32 fishers had insufficient tissues for a full necropsy and were classified as predation events via molecular forensics and/ or ante-mortem hemorrhaging from wounds on remaining tissues. Specific predators of fishers could be determined for 67% (n = 60) of all predation events based on molecular forensic evidence; eight more were identified only to family, specifically Felidae. Of predators identified, bobcats (*Lynx rufus*, n = 27: 40%), mountain lions (*Puma concolor*, n = 26: 38%), unidentified Felidae (n = 8: 12%), coyotes (*Canis latrans*, n = 4: 6%), and domestic dogs (*Canis lupus familiaris*, n = 2: 3%) were confirmed predators of fishers while a single fisher (1%) was killed by a rattlesnake (*Crotalus oreganus oreganus*).

### Natural disease

Of the 21 mortality events for collared fishers attributed to natural disease, 48% (n = 10) were attributed to bacterial infections, 28% (n = 6) to emaciation, 14% (n = 3) to viral infections, 5% (n = 1) to a protozoal infection and 5% (n = 1) to malignant neoplasia (cancer). Of the 10 bacterial infections, nine were associated with interstitial pneumonia or bronchopneumonia. Three of the four northern California mortalities due to bacterial infection had a concurrent, nematode parasitism of the lungs, which was not identified to genera. The nematode parasitism cases were associated with interstitial pneumonia with bacterial infiltrates, although this was not the proximate cause of mortality. Four of the six fisher mortalities due to bacterial infection in the southern Sierra Nevada had bacterial infiltrates associated with interstitial pneumonia, but contamination with mixed bacterial flora prevented identification. Two of these cases also involved an unknown lung nematode. The remaining two cases were septic with mixed bacterial flora, which may have resulted from cutaneous punctures with associated necrosis and bacterial infiltration consistent with predator bite wounds.

All six fishers that died due to starvation were severely emaciated with no pericardial, renal, mesentery or subcutaneous fat. All of these cases showed emaciation with no other detectable concurrent disease processes. For five of the six emaciation cases, the cause for emaciation was unknown. The remaining case was a female fisher with an acute complete fracture of the left mandible coupled with numerous canine, incisor and molar teeth fractures. The source of this acute trauma was unknown however predation, an illegal snare or vehicular strike, though presumptive, may have been the contributing cause due to the force required. In addition, two altricial kits were recovered from abandoned den sites and determined to have died of emaciation.

All three fishers that died of a viral etiology were infected with CDV as previously described [18]. We categorized one fisher as predation mortality that had a concurrent systemic CDV infection. The lesions caused by CDV were widespread and severe suggesting that they had a debilitating clinical effect, which facilitated the predation event. The mortality was hence classified as ‘predation’ rather than ‘disease’, though this fisher most likely would have succumbed to distemper due to the systemic infection. The sole mortality attributed to protozoal infection was due to a severe, non-suppurative, menigioencephalomyelitis caused by *T*. *gondii* as determined by immunohistochemistry. The only fisher that died from cancer had systemic lymphoma involving lymph nodes, liver and skin.

### Poisoning

Thirteen fishers in the two populations died of toxicosis, all of which had trespass marijuana (*Cannabis sativa*) cultivation and associated toxicants within their home ranges. Anticoagulant rodenticides (ARs), which are toxicant compounds that inhibit the recycling of vitamin K1 leading to clotting and coagulation impairment, caused 11 fisher mortalities. In addition to detection of AR in these fishers’ livers, they exhibited coagulopathy and significant hemorrhage. Exposure to ARs alone did not constitute an AR toxicosis case. In addition to ARs, cholecalciferol, another rodenticide which causes hypercalcemia and has been found at several cultivation sites in the northern California project, was assumed to be the contributing cause of death in one male fisher from northern California. This fisher had multifocal mineralization in the aorta, testes and renal medulla. All other causes of hypercalcemia such as chronic renal failure and hyperparathyroidism were ruled out and cholecalciferol rodenticides were discovered near this fisher’s home range. The kidneys for this fisher were submitted for total vitamin D_3_ assay (a measure to detect Vitamin D toxicosis) along with another kidney from a fisher that exhibited no mineralization in any tissues (Heartland assays LLC, Ames, Iowa, USA). Results demonstrated a 7.4 fold difference of total vitamin D_3_ between the two samples (14.1 ng/g vs. 1.9 ng/g). Unfortunately, sample identifications during laboratory submission were not legible to lab staff, therefore correct assignment of results to the sample could not be completed with confidence. This fisher was also exposed to five different ARs, for a total of six different rodenticides it had consumed.

Another collared male fisher from northern California exhibited neurological signs including ataxia, lethargy and seizures before being euthanized (Permanent Video Link: https://www.youtube.com/watch?v=otognB4LdTY). This fisher was near an illegal marijuana cultivation site where bromethalin and carbamate insecticides, as well as numerous organophosphates, were found. However, no carbamates, organophosphates, illicit drugs, metaldehyde or bromethalin were detected in the stomach contents, liver, urine or kidney. In addition, we tested its bile for Anatoxin-a, but did not detect it in the sample. All other potential mechanisms for this fisher’s clinical signs were ruled out leading this case to be classified as suspected toxicosis.

Seven of the toxicosis cases were from the northern California population while the remaining 6 were from the southern Sierras (Table 2). Annual fisher mortality attributed to rodenticides varied with an average of 1.86 toxicosis cases each year (2007:0, 2008:1, 2009:1, 2010:3, 2011:0, 2012:3, 2013:4, 2014:1). Nine of the 13 toxicosis cases occurred in spring (March-June: 69%), three in late winter (February: 23%) and one in fall (October: 8%). A total of 101 fishers had sufficient liver tissue to test for anticoagulant rodenticide exposure. Of these fishers, 86 (85%) were exposed to one or more ARs and had an average of 1.73 different AR compounds (range: 1–5, SD:0.91).

### Vehicle strikes

All 10 fishers killed by vehicular strike were discovered on paved road systems with various speed limits for vehicles. Seven of these were uncollared and opportunistically collected, whereas three road killed fishers from the southern Sierra Nevada population had been radio-collared. Two additional fisher mortalities from the southern Sierras were originally suspected to be vehicular strikes due to the carcasses being discovered near or on a roadway but had no evidence of blunt force trauma, macerated muscles, comminuted fractures, torn viscera or ruptured blood vessels, all of which were observed in all of the 10 confirmed vehicular strike cases. These fishers were finally ruled as AR poisoning due to the significant pleural and abdominal cavity hemorrhaging, in addition to several ARs detected in tissues.

### Anthropogenic causes

The two cases of human-caused mortalities were due to entrapment in man-made structures. A radio-collared female adult fisher at HVRFP died of dehydration when she was caught in a live trap that was inadvertently left operational between trapping sessions. The maximum duration over which the fisher could have been left in the live trap was five days. The second fisher was an uncollared, sub-adult male that was discovered in an air quality sampling tube at the KRFP study. This fisher’s tissues were too autolyzed to perform a necropsy. A third fisher from the southern Sierras was initially suspected to have died from negative reaction from recalled ketamine. However upon necropsy, it was determined that this fisher was infected with CDV exhibiting clinical signs of disease but respiratory depression from anesthesia was the proximate factor which expedited inevitable death due to CDV infection [18].

### Field-based vs. necropsy-confirmed causes of mortality

Of the 136 fisher carcasses for which cause of mortality was identified, field biologists reported cause of mortality as predation for 66% (n = 90) of fisher deaths, “unknown” for 12% (n = 16), disease for 8% (n = 11), vehicular strike for 9% (n = 12), other human-caused for 4% (n = 6) and drowning for 1% (n = 1) (Table 3). Three suspected human-caused mortalities were subclassified as “delayed negative anesthesia-reaction” and one was classified as a “VHF collar hanging”. The kappa statistic for test agreement between biologist-determined cause of death and necropsy-confirmed cause of death was 0.5669, showing only moderate agreement. In contrast to the field-based suspected causes, pathological investigation indicated no mortalities were attributed to drowning, collar strangulation, or negative reactions to anesthesia. All of these mortalities were caused by disease. Disease was the mortality cause most underestimated by field biologists, while predation and vehicular strike were overestimated (Table 3).

**Table 3:** Field-based causes of mortality determined by field evidence alone and necropsy determined causes of mortality for fishers (*Pekania pennanti*) within the two isolated populations, northern California and southern Sierra Nevada. Data were combined from 2007 to 2012 (n = 136).

			Necropsy Determined Causes of Mortality					
Field-based Suspected Cause of Mortality	Predation	Disease	Poisoning	Human	Vehicular	Drowning	Unknown	Total
Predation	**86**	3	1	0	0	0	0	90
Disease	0	**4**	5	0	0	0	0	9
Poisoning	0	0	**2**	0	0	0	0	2
Human	0	4	0	**2**	0	0	0	6
Vehicular	0	0	2	0	**10**	0	0	12
Drowning	0	1	0	0	0	**0**	0	1
Unknown	4	9	3	0	0	0	**0**	16
Total	90	21	13	2	10	0	0	**136**

Kappa = 0.5669.

### Multinomial logistic regression

The final fitted multinomial logistic regression model assessing the association of demographic data with cause of mortality identified sex and population as significant independent predictors of the cause of mortality (Table 4). No other variables were significant. In the comparison of poisoning vs. other causes of mortality, sex was the most significant predictor affecting cause of mortality (Table 5). Compared to females, males were approximately 7 times more likely to die of poisoning than natural disease (OR = 6.9, 95% CI: 1.19–40.18, p = 0.0313) and 13 times more likely to die of poisoning than predation (OR = 13.04, 95% CI: 2.59–65.69, p = 0.0019). Fishers from the northern California population were almost 5 times more likely than southern Sierra fishers to die of rodenticide than predation (odds ratio = 0.21, 95% CI: 0.06–0.77, p = 0.018).

**Table 4:** Performance statistics of three top models of demographic factors relating to ultimate cause of mortality for 136 fishers (*Pekania pennanti*) within the two isolated populations, northern California and southern Sierra Nevada. The two factors in the final model were SEX (sex of the fisher) and POPN (population of fisher).

Model	K	Log-likelihood	AICc	∆AICc	*w*_*i*_
SEX	6	-129.634	271.919	0.000	0.523
SEX + POPN	9	-126.433	272.294	0.375	0.433
NULL	3	-136.040	278.262	6.343	0.022

**Table 5:** Results of a multinomial logistic regression in the final model indicating the effects of fisher (*Pekania pennanti*) sex and population on likelihood of mortality from a specific cause. Significant variables in the model are bolded.

		Rodenticide vs. Disease						Rodenticide vs. Human-caused						Rodenticide vs. Predation					
				Odds	95%CI	95%CI				Odds	95%CI	95%CI				Odds	95%CI	95%CI	
Characteristic		Coeff	SE	Ratio	Lower	Upper	p-value	Coeff	SE	Ratio	Lower	Upper	p-value	Coeff	SE	Ratio	Lower	Upper	p-value
*Intercept*		-1.31	0.83	NA	NA	NA	0.113	-0.60	0.916	NA	NA	NA	0.516	-2.57	0.764	NA	NA	NA	0.0008
*Population*																			
	NC (reference)	-	-	-	-	-	-	-	-	-	-	-	-	-	-	-	-	-	
	SS	-0.883	0.743	0.414	0.10	1.77	0.234	-1.08	0.852	0.34	0.06	1.8	0.205	-1.56	0.659	0.21	0.06	0.77	**0.018**
*Sex*																			
	Female (reference)	-	-	-	-	-	-	-	-	-	-	-	-	-	-	-	-	-	
	Male	1.933	0.898	6.912	1.19	40.18	**0.031**	1.87	0.978	6.46	0.95	43.89	0.564	2.57	0.825	13.04	2.59	65.69	**0.002**

The multinomial logistic regression model assessing the association between anticoagulant rodenticide or pathogenic exposure factors and causes of mortality indicated that the number of individual AR compounds to which a fisher was exposed was associated with cause of mortality (Table 6). For every additional AR type to which a fisher was exposed, its likelihood of dying of poisoning vs. disease increased almost 3-fold (OR = 2.75, 95% CI: 1.25–6.07, p = 0.0122) and of dying of poisoning versus predation increased approximately by 2.5 (OR = 2.56, 95% CI: 1.25–5.25, p = 0.0105) (Table 7). Fishers were almost 4 times as likely to die of poisoning vs. human-caused with each additional AR type to which they were exposed (OR = 3.75, 95% CI: 1.29–10.86, p = 0.0150). Exposure to the three pathogens was not a significant predictor of causes of mortality.

**Table 6:** Performance statistics of three top models of pathogen and toxicant exposure factors relating to ultimate cause of mortality for 67 fishers (*Pekania pennanti*) within the two isolated populations, northern California and southern Sierra Nevada. The one factor in the final model was ARNUM (number of different types of anticoagulant rodenticides to which the fisher was exposed). TOXO_high refers to exposure to *T*. *gondii* using the isotype IgM.

Model	K	Log-likelihood	AICc	∆AICc	*w*_*i*_
ARNUM	6	-76.628	170.656	0.000	0.606
TOXO_high + ARNUM	9	-76.491	174.141	3.485	0.106
NULL	3	-84.073	174.527	3.871	0.088

**Table 7:** Results of a multinomial logistic regression in the final model indicating the effects of the number of different types of anticoagulant rodenticides (AR) a fisher (*Pekania pennanti*) was exposed to on likelihood of mortality from a specific cause. Significant variables in the model are bolded.

	Rodenticide vs. Disease						Rodenticide vs. Human-caused						Rodenticide vs. Predation					
			Odds	95%CI	95%CI				Odds	95%CI	95%CI				Odds	95%CI	95%CI	
Characteristic	Coeff	SE	Ratio	Lower	Upper	p-value	Coeff	SE	Ratio	Lower	Upper	p-value	Coeff	SE	Ratio	Lower	Upper	p-value
*Intercept*	-2.59	0.92	NA	NA	NA	**0.005**	-2.11	1.033	NA	NA	NA	**0.041**	-2.94	0.882	NA	NA	NA	**0.0008**
*AR Number*	1.01	0.404	2.75	1.25	6.07	**0.012**	1.32	0.543	3.75	1.29	10.86	**0.015**	0.94	0.367	2.56	1.25	5.25	**0.011**

## Discussion

This study is the first to thoroughly describe necropsy-confirmed, cause-specific mortality of fishers in the West Coast DPS and our findings provide baseline information on the mortality factors potentially limiting fisher populations in other portions of this DPS [8, 35, 36, 37]. The most significant findings of our study were the relative importance of predation and poisoning as mortality factors and the apparent increase of pesticide poisoning frequency in a short span of time. Importantly, our finding that AR poisoning was a more likely cause of death than predation in the northern California population versus the southern Sierra Nevada signifies regional heterogeneity in anthropogenic influences in forest landscapes. Besides differences in likelihood of AR poisoning, we found little heterogeneity in most causes of mortality between the two study populations or among years supporting their generality in California fisher populations. This finding likely reflects similarities in habitat, prey utilization, and predator communities throughout the range of fishers in California [2, 3, 38]. A secondary finding was that field assessment of cause of death significantly underestimated the frequency of natural disease-related mortalities.

Our results confirmed earlier findings that predation was a significant mortality factor affecting fishers in California, causing the majority of all fisher deaths [19, 26, 35]. The addition of 28 new predation cases for this study did not change the frequencies of predation events by particular predator species for fishers from both populations determined in an earlier study [35]. Older studies suggested that predation was an insignificant mortality factor, thought primarily to affect vulnerable or reintroduced individuals [2, 13, 39]. In our study, females more frequently died from predation (relative to other causes) than males, possibly attributable to the smaller mass of females. Female fishers on average were half to two-thirds the mass of males [13], thus potentially increasing their susceptibility to a greater diversity of predators. Additionally, the importance of predation becomes more clear in light of recent findings suggesting that population size for fisher is heavily influenced by adult female survival [8]. It is unclear why these isolated California populations were subject to such high prevalence of predation relative to other populations, but further investigation of this finding is critical to the conservation of the California populations [8, 13, 39–41]. Furthermore, we do not know whether the predation rates we observed for fishers are different from predation rates that fisher populations have suffered throughout their evolutionary history. However, recent research into the effects of habitat modification on likelihood of fisher predation does suggest that changes in habitat over the past century may be changing the rate of predation on female fishers by bobcats [42].

Although exposure to the protozoan *T*. *gondii* has been shown to predispose individuals to predation or vehicular strikes [23, 24, 43], we found no significant evidence of this relationship. However, many of the depredated fishers did not have any available blood to sample due to the predator consuming the heart or exposing the thoracic cavity leaving unsuitable samples for testing, resulting in a small sample size with which to detect such a relationship. Then again, all available brain tissue was tested for gliotic foci due to *T*. *gondii* and none were found.

Natural disease was the second-most frequent cause of mortality in our study. Kits died from disease more frequently than any other cause, likely since kits were den-bound and therefore less exposed to predators and humans. Bacterial infections accounted for the largest number of disease-related fisher deaths, and generally manifested as bacterial pneumonia. However, in no instance did we identify a single dominant bacterial pathogen but cultures yielded mixed flora in all cases. These results may be due to post-mortem autolysis and contamination of pathogenic bacteria by opportunistic species. Interestingly, two of the mortalities associated with bacterial infection also had full thickness, circular punctures in the skin suggestive of failed predation attempts resulting in a site for introduction of a bacterial infection. It should be noted that pulmonary viral infections that might have preceded and facilitated bacterial colonization could not be identified but cannot be ruled out.

The toxicosis cases discovered in this study signify an increase of this emerging threat for fishers in the West Coast DPS [12, 36]. Cultivation of marijuana and the associated use of toxicants have been recently documented in occupied fisher habitat [12, 36, 44]. In addition to the four fisher mortalities attributed to anticoagulant rodenticides by Gabriel et al. (2012), we documented nine additional pesticide toxicosis cases in the present study. The average incidence of toxicosis cases per year for the five year Gabriel et al. (2012) study spanning 2007–2011 was 5.6% (SE = 3.1%). However, in the final three years (2012–2014) of our study, we detected an increase in incidence per year to 18.7% (SE = 2.9%). Exposure also increased from 79% (46 of 58) to 85% (86 of 101) for the same two time periods [12]. This increase in cases and exposure could signify either an increase in the number of cultivation sites or area impacted or that cultivators are increasing the level of toxicants being dispersed within occupied fisher home ranges. In either case, this anthropogenic threat is of increasing concern.

Previous reports of cholecalciferol poisonings have not been reported in a remote forest dwelling carnivore. This type of toxicant has been promoted as an alternative to anticoagulant rodenticides due to the minimized risk for secondary poisonings [45]. Nevertheless, plant and animal based food flavorizers are often incorporated into rodenticides to enhance palatability to omnivorous rodents [12]. Because fishers are omnivorous [2], they could be susceptible to primary poisoning if they are attracted to these compounds when they are impregnated with flavorizers. In addition, the massive amount of rodenticide dispersed at some cultivation sites e.g>40 kg in some sites, which have cultivation footprints of typically less than 0.2ha [12, 46] likely pose a secondary risk of poisoning to fishers. Fishers may consume numerous prey that may have recently ingested these rodenticides, with the likely exception of cholecalciferol.

As was noted previously for a subset of cases, toxicosis deaths occurred primarily in the spring [12]. Additionally, males were more likely than females to die of poisoning relative to predation and other causes. This finding may be due to fewer predation events involving males than females [19, 35] or the higher prevalence of poison-related mortality in males. These trends could also be due to behavioral factors [28]. Female fishers in California increase their crepuscular and diurnal activity in spring to satisfy the additional energy requirements of lactation and care of weaned kits but typically within the confines of their established home-ranges. Male fishers may make extensive forays outside their normal home ranges in spring to search out females for mating opportunities [2, 13]. Marijuana cultivation coincides with the increased activity of fishers in early spring and frequently involves dispersal of large amounts of toxicants near occupied fisher home ranges [12, 36, 46]. Furthermore, survival of female fishers in one population was found to be influenced by the number of marijuana cultivation sites in the 95% fixed kernel home range [36].

The relationship between the number of ARs to which a fisher has been exposed and the increasing probability of death due to poisoning suggests that these pesticides may be acting additively or synergistically. However, little experimental data are available demonstrating exposure to multiple ARs increasing the risk of coagulopathy, [12, 36, 47, 48]. Our data suggest that coagulopathy risk increases significantly with each additional new AR compound exposure, though it’s possible this pattern is reflecting an additive relationship between AR number and cumulative level of exposure. However, potential synergistic mechanisms need to be addressed due to the significant amount of other pesticides, herbicides, molluscicides and fungicides documented at marijuana cultivation sites. Because fishers are exposed to > 1.7 different ARs on average, our concerns on the potential unknown mechanisms of deleterious effects of multiple ARs warrants further investigation [36, 46, 47].

Human-related mortalities were relatively rare, and although a small number were associated with research activities, such mortalities represented < 1% of the captured fishers. This figure is comparable to other studies [49]. Vehicle-related mortalities were also relatively rare with only three marked fishers suffering vehicle strikes, which represented < 2% of all mortalities. The higher number of uncollared fishers found killed in roadways suggests that roadkill may be a more local concern, associated with individual high-traffic corridors.

Field biologists did not always accurately identify general causes of disease. We found only a moderate correspondence between biologist-determined and necropsy-confirmed causes of death except for the detection of disease-related mortalities, which were significantly underestimated by initial field assessments. For example, the three fisher deaths attributed to CDV and many of the toxicosis cases were preliminarily attributed to other causes in the field. The underestimation of disease has been observed in other wildlife studies because gross observations in the field are inadequate to detect subtle signs of disease [18]. These findings fortify the need for full necropsies when studying causes of mortality, especially when knowledge of the frequencies of cause-specific mortality is required in managing or reducing the most significant limiting factors for fishers.

Although predation was often correctly identified by both field biologists and the pathologists, the incorporation of molecular forensic approaches coupled with traditional pathology allowed us to more definitively identify both predation events and predator species [19, 26, 35]. Predation is often implicated as the cause of mortality when field evidence such as tracks near or adjacent to the carcass, bite wounds, wound patterns or feces and/or hair near the carcass are found [9, 50–53]. However in our study, field observations misclassified 5 fishers as predation due to circumstantial predator evidence found near the carcass (e.g. tracks, scat). Field observations can be misleading, for example, bite wounds in soft tissue often change shape and size due to environmental factors [26, 54] (Linda Munson, University of California Davis, Personal Communications) and visual artifacts that resemble ante-mortem hemorrhaging can occur due to autolysis, scavengers consuming tissue and releasing non-clotted blood, or freezing and defrosting of a carcass.

Finally, we present mainly the proximate causes of mortality for fishers though there were a few cases where ultimate causes could be ascertained e.g. anesthesia related death but clinically infected with CDV. However, it would be difficult, if not impossible, to determine whether some of the predation mortalities were ultimately going to result in toxicosis. Many of the predation cases exhibited ante-mortem hemorrhaging that could have been due directly to predation or alternatively, AR exposure. Anticoagulant rodenticides have previously been shown to cause lethargy and weakness in exposed animals [12, 47], but teasing these two causes of death apart was not possible.

This study presents the first large assessment of cause-specific mortality frequencies in California fishers. We have identified predation and natural disease as the top two mortality factors. In addition, mortality from and exposure to toxicants appears to be on the rise and we have found exposure to multiple ARs increases probability of death from these compounds. Increases of additive mortality of only 10% can prevent fisher population expansion even in the presence of suitable habitat with no dispersal barriers [8]. Therefore, the high proportion of fisher mortality consisting of predation and disease may help explain the lack of growth and expansion of these populations to nearby suitable habitat. However, the growing number of toxicosis cases in fishers and the correlation of contributing mechanisms such as marijuana cultivation within fisher habitat suggest an emerging threat. Beyond direct poisoning, rodenticides have the potential to limit fitness through prey depletion and heightened competition between fishers and other carnivores. Future research should focus on the relationship between marijuana cultivation and associated rodenticide use and prey population cycles because carnivore population dynamics are often heavily influenced by fluctuations in prey base [55, 56].

Managing these threats should focus not only on the impacts on current fisher populations but also the reduction of threats that may be limiting expansion for future population growth. One recommendation is the complete removal of toxicants left at current and historicaltrespass marijuana grow sites. Most sites are not remediated, thus toxicants associated with these sites are a continuing threat. Furthermore, as female adult survival is notably important for population size and persistence in the southern Sierra Nevada population, forest managers should consider managing against habitat features that are conducive to interactions between fishers and their predators. Investigating these and other mechanisms for reducing mortality in California fishers within West coast DPS can be of assistance in effectively implementing policy or management options to potentially curb mortality rates in order to promote population recovery within California in addition to other fisher populations throughout the West Coast DPS.
